# *Schizophyllum commune*: An unexploited source for lignocellulose degrading enzymes

**DOI:** 10.1002/mbo3.637

**Published:** 2018-05-21

**Authors:** Omar Eduardo Tovar‐Herrera, Adriana Mayrel Martha‐Paz, Yordanis Pérez‐LLano, Elisabet Aranda, Juan Enrique Tacoronte‐Morales, María Teresa Pedroso‐Cabrera, Katiushka Arévalo‐Niño, Jorge Luis Folch‐Mallol, Ramón Alberto Batista‐García

**Affiliations:** ^1^ Instituto de Biotecnología Facultad de Ciencias Biológicas Universidad Autónoma de Nuevo León Ciudad Universitaria San Nicolás de los Garza Nuevo León México; ^2^ Laboratorio de Micología y Fitopatología Unidad de manipulación genética Facultad de Ciencias Biológicas Universidad Autónoma de Nuevo León Ciudad Universitaria San Nicolás de los Garza Nuevo León México; ^3^ Centro de Investigación en Biotecnología Universidad Autónoma del Estado de Morelos Cuernavaca Morelos México; ^4^ Instituto del Agua Universidad de Granada Granada Granada Spain; ^5^ Universidad Central del Ecuador Quito Ecuador; ^6^ Centro de Investigación en Dinámica Celular Universidad Autónoma del Estado de Morelos Cuernavaca Morelos México

**Keywords:** biorefinery, biotechnology, lignocellulolytic enzymes, lignocellulose, *Schizophyllum commune*

## Abstract

Lignocellulose represents the most abundant source of carbon in the Earth. Thus, fraction technology of the biomass turns up as an emerging technology for the development of biorefineries. Saccharification and fermentation processes require the formulation of enzymatic cocktails or the development of microorganisms (naturally or genetically modified) with the appropriate toolbox to produce a cost‐effective fermentation technology. Therefore, the search for microorganisms capable of developing effective cellulose hydrolysis represents one of the main challenges in this era. *Schizophyllum commune* is an edible agarical with a great capability to secrete a myriad of hydrolytic enzymes such as xylanases and endoglucanases that are expressed in a high range of substrates. In addition, a large number of protein‐coding genes for glycoside hydrolases, oxidoreductases like laccases (Lacs; EC 1.10.3.2), as well as some sequences encoding for lytic polysaccharide monooxygenases (LPMOs) and expansins‐like proteins demonstrate the potential of this fungus to be applied in different biotechnological process. In this review, we focus on the enzymatic toolbox of *S*. *commune* at the genetic, transcriptomic, and proteomic level, as well as the requirements to be employed for fermentable sugars production in biorefineries. At the end the trend of its use in patent registration is also reviewed.

## 1 INTRODUCTION

In the past decade, the amount of research related to lignocellulosic ethanol (second generation ethanol) has increased extensively in the scientific community. Several bacteria and fungi species have been studied in terms of the intra and extracellular enzymatic complexes involved in the deconstruction of the polymeric components that make up the lignocellulosic biomass. On the other hand, every year a novel or modified pretreatment technology becomes available with the aim of improving yields during the saccharification of the lignocellulosic materials. However, we are still far away from producing economically competitive lignocellulosic bioethanol (Mohanram, Amat, Choudhary, Arora, & Nain, 2013), largely because the lack of microbial enzymatic cocktails that break down the recalcitrant lignocellulosic biomass in an efficient manner (Gupta, 2016). Since the amount of plant biomass has been estimated to be of 180 billions of tons only above the ground and near 40 millions tons in the ocean (Chen, 2014), the exploitation of these materials for production of biofuels and value‐added products is a great alternative to reduce the fossil fuels dependence that as a society we have.

This review focuses on the unexploited and enormous biotechnological potential of the basidiomycete fungus *Schizophyllum commune* for the production of novel enzymes that could boost the biofuel and biomass derived product research. Also, this work summarizes the research that has been conducted in the last two decades and that supports the use of *S*. *commune* as a current aspirant for white, green and gray biotechnology applications.

## 2 GENERAL ASPECTS OF *SCHIZOPHYLLUM COMMUNE*

**Figure 1:**
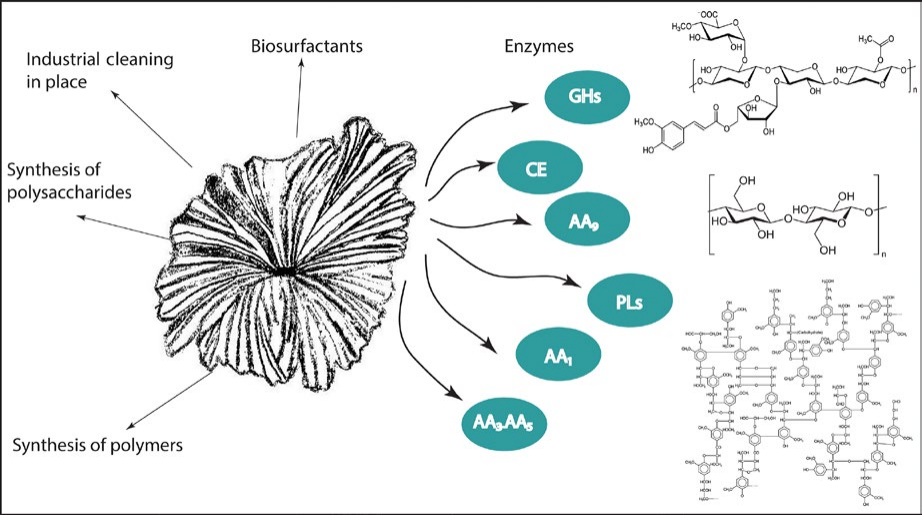
Biotechnological applications of *Schyzophyllum commune*. Glycoside hydrolase (GH), carbohydrate esterase (CE), glycosyltransferase (GT), polysaccharide lyase (PL), lytic polysaccharide monooxygenase (AA9), laccase (AA1), peroxide‐producing enzymes (AA3, and AA5)

**Figure 2:**
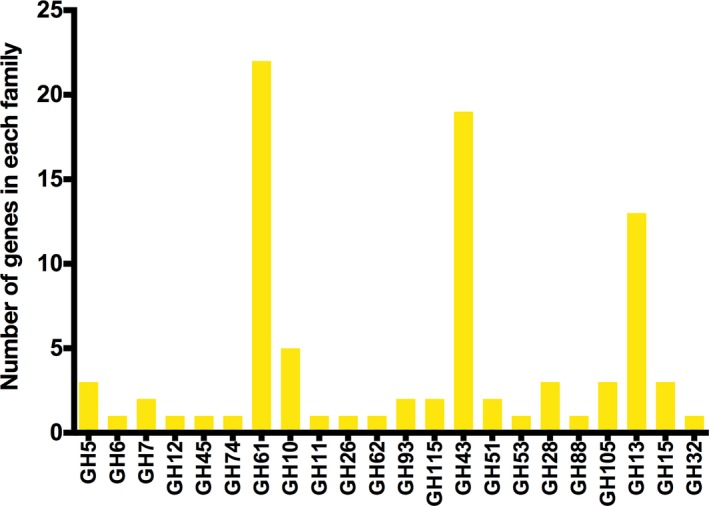
Glycoside hydrolase (GH) genes present in the genome of *Schizophyllum commune*. Only those involved in plant cell wall deconstruction were considered

*Schizophyllum commune* is an agarical mushroom‐forming fungus, able to complete its life cycle in about 10 days and is one of the most commonly found fungi, whose distribution covers all continents with the exception of Antarctica (Ohm, de Jong, Lugones, et al., 2010). *Schizophyllum commune* has been successfully genetically modified and used as molecular tool for studying cell wall biogenesis (Wessels, 1986), hyphal fusion and development (Ahmad & Miles, 1970; Van Wetter, Schuren, Schuurs, & Wessels, 1996), mating type (Kothe, 1999; Yang, Shen, Park, Novotny, & Ullrich, 1995), heterologous expression of genes (Schuren & Wessels, 1998), gene deletions (De Jong, Ohm, De Bekker, Wösten, & Lugones, 2010; Ohm, de Jong, Berends, et al., 2010), among others. Although it has been detected causing illness in animals and humans, its lifestyle is mainly saprobic by causing white rot. Actually, it has been reported that at least 150 genera of woody plants are substrates for *S*. *commune*, but it also colonizes softwood and grass silage (Ohm, de Jong, Lugones, et al., 2010). This feature is one of the most interesting points in a biotechnological sense about this fungus, since it allows *S*. *commune* to colonize a vast diversity of lignocellulosic substrates, expanding the range of possibilities and biotechnological products (e.g., enzymes (phytase, lipase, holocellulase, etc.) (Arboleda Valencia et al., 2011; Salmon et al., 2012; Singh, Singh, Kumar, & Thakur, 2015), bioethanol (Horisawa, Ando, Ariga, & Sakuma, 2015), biosurfactants (Wessels, de Vries, Asgeirsdottir, & Springer, 1991), industrial cleaning‐in‐place (CiP) agents (Boyce & Walsh, 2012), polysaccharides (Singh, Kumar, & Thakur, 2017), polymers (Jayakumar, Kanth, Chandrasekaran, Raghava Rao, & Nair, 2010), etc.) that can be obtained with this microbe (Figure 1). As a matter of fact, *S*. *commune* has the potential to degrade all components of the lignocellulosic biomass, since its genome contain 240 gene candidates for glycoside hydrolases (89 account for plant polysaccharides degradation, see Figure 2), 75 for glycosyl transferases, 16 for polysaccharide lyases, 17 for expansin‐related proteins, 30 for carbohydrate esterases, and 16 for lignin‐degrading oxidoreductases (Ohm, de Jong, Lugones, et al., 2010). This extensive repertoire of plant cell wall degrading and modifying enzymes makes *S*. *commune* an outstanding candidate for studies regarding the mechanism by which this fungus degrades biomass in order to exploit its potential and improve the efficiency of industrial processes such as the lignocellulosic ethanol production, bioconversion of agricultural by‐products or biodegradation of xenobiotics and pollutants (Table 1).

**Table 1:** Examples of biotechnological uses of *Schizophyllum commune*

Biotechnological uses	References
Lipase production	Singh, Singh, Kumar, and Thakur (2014)
Phytase production	Salmon et al. (2012)
Lipase inmobilization for fatty acids methyl esters	Singh et al. (2015)
Decolorization of textile dyes	Asgher, Yasmeen, and Iqbal (2013) and Bhatti, Akram, and Asgher (2008)
Decolorization of Azo dyes and synthetic dyes	Tang, Jia, and Zhang (2011) and Yao, Jia, Zheng, and Wang (2013)
Biosorption of heavy metals	Amna, Bajwa, and Javaid (2010), Gabriel, Švec, Kolihová, Tlustoš, and Száková (2016), Javaid and Bajwa (2008)
Biotransformation of sophorocoside	Wu et al. (2012)
Direct ethanol production	Horisawa et al. (2015)
Holocellulase production	Arboleda Valencia et al. (2011)
Lignocellulose degradation	Asgher et al. (2016)
Phenolic compounds biosorption	Kumar and Min (2011)
Schizophyllan production	Kumari, Survase, and Singhal (2008)
Polysaccharide derived antimicrobials	Jayakumar et al. (2010)

## 3 PROTEINS INVOLVED IN CELLULOSE DECONSTRUCTION BY *SCHIZOPHYLLUM COMMUNE*

The subject of cellulose deconstruction by fungi leads us to think immediately of organisms like *Trichoderma reesei*,* Neurospora crassa*, and various *Aspergillus* species, considering the ascomycetes group, and mainly in *Phanerochaete chrysosporium* when referring to the basidiomycetes group, leaving out of study a significant group of basidiomycetes with the same or even greater potential to degrade cellulose. One of these basidiomycetes is the “split gill” fungus *S*. *commune*, whose genome sequence was published in 2010 (Ohm, de Jong, Lugones, et al., 2010). Its genome revealed that it contains 240 candidate genes belonging to glycoside hydrolases from different families, almost 80 more GH genes than those reported for *P. chrysosporium*.

The study of the hydrolytic machinery in organisms like *S*. *commune* is attractive mainly for the lignocellulosic biofuels industry, since the number of published works is still growing year by year in this subject, but is not limited to this area. Something remarkable is that the amount and diversity of published work related with the *S*. *commune* ′s cellulolytic system is scarce (Table 2) when compared with published work (in this area) of fungi like *T*. *reesei*,* N*. *crassa* or *P*. *chrysosporium*, despite the fact that studies such as those carried out by Arboleda Valencia et al. (2011), Lee et al. (2014), Zhu et al., (2016) have demonstrated that *S*. *commune* has an important potential in the lignocellulose bioconversion field, even exhibiting cellulolytic and xylanolytic activities comparable with those obtained using an enzymatic commercial preparation of *Trichoderma longibranchiatum*.

**Table 2:** Cellulolytic and xylanolytic enzymes studied in *S*. *commune*

Enzyme	Inducer substrate	References
β‐Glucosidase	Cellulose	Desrochers, Jurasek, and Paice (1981)
Endoglucanase β‐glucosidase	Thiocellobiose	Rho, Desrochers, and Jurasek (1982)
Xylanase	CMC	
	Cellobiose	
	Xylan	
Endoglucanase	Cellulose	Willick and Seligy (1985)
β‐Glucosidase		
Xylanase		
Endoglucanase	Unknown	Clarke and Adams (1987)
Xylanase	Avicel	Steiner, Lafferty, Gomes, and Esterbauer (1987)
Endoglucanase		
Xylanase	Bacteria cellulose	Haltrich, Sebesta, and Steiner (1996)
Endoglucanase	Cellobiose	
	Sophorose	
	Birchwood xylan	
Acetylxylan esterase	Unknown	Biely et al. (1996)
Cellulase GH5	Unknown	Clarke, Drummelsmith, and Yaguchi (1997)
α‐Glucuronidase	Cellulose	Tenkanen and Siika‐Aho (2000)
	Wheat bran	
	Distiller′s spent grain	
Xylanase	Cellulose	Kolenová, Vršanská, and Biely (2005)
Glucuronyl esterase	Cellulose	Špániková and Biely (2006)
Xylanase	Bamboo fibers	Arboleda Valencia et al. (2011)
Mannanase	Banana stem	
Polygalacturonase	Sugarcane bagasse	
Endoglucanase		
Fpase		
Avicelase		
α‐Glucuronidase	Recombinant	Chong et al. (2011)
Xylanase	Cellulose	Tsujiyama and Ueno (2011)
CMCase	Rice straw	
β‐Glucosidase	Wood	
Acetylesterase		
Cinnamic acid esterase		
β‐Glucosidase	Cellulose	Lee et al. (2014)
Avicelase	Avicel	Luziatelli et al. (2014)
FPase	Tamarix leaves	
β‐Glucosidase		
α‐Amylase		
Expansin	Recombinant	Tovar‐Herrera et al., (2015)
Endoglucanase	Jerusalem artichoke stalks	Zhu et al. (2016)
Cellobiohydrolase		
β‐Glucosidase		
α‐Glucuronidase	Recombinant	McKee et al. (2016)
β‐Glucosidase	Cellulose	Lee et al. (2017)
Feruloyl esterase	Recombinant	Nieter, Kelle, Linke, and Berger (2016)

The cellulose degradation mechanisms by ascomycetes and basidiomycetes have been revised by Glass, Schmoll, Cate, and Coradetti (2013) and Baldrian and Valásková (2008). However, although the role of the classic enzymes involved in cellulose deconstruction such as endoglucanases, cellobiohydrolases, cellobiose dehydrogenases and beta glucosidases is well documented in fungi, the role of the termed “amorphogenic proteins” or plant cell wall remodeling proteins (expansins and expansin‐related proteins) in cellulose deconstruction is not well understood in both, ascomycetes and basidiomycetes. These amorphogenic proteins cause swelling of cellulose fibers and fragmentation of cellulose aggregations at the beginning of the enzymatic hydrolysis of cellulose before any detectable amount of reducing sugars is released (Gourlay et al., 2013). From these latter proteins, the swollenin from *T*. *reesei* is the most studied, and after its discovering it was suggested as the *C*_1_ factor of the cellulose enzymatic degradation mechanism originally proposed by Mandels and Reese (1999) and Reese, Siu, and Levinson (1950). Nevertheless, that hypothesis has been rejected by the work of Eibinger et al. (2016), who demonstrates that swollenin is not an amorphogenesis factor when acting on pure cellulose. Nonetheless, the possibility that one or more of these plant cell wall remodeling proteins may be acting as the C_1_ factor is yet to be proven. Indeed, the genome of *S*. *commune* contains at least 17 expansin‐related proteins, one of which has already been cloned and expressed in *Pichia pastoris*, showing a 23% increment in avicel hydrolysis when used as pretreatment before the addition of a cellulase mixture (Tovar‐Herrera et al., 2015). However, the study of this type of proteins is relatively new in microbes, and there is a lot of information yet to be obtained from them.

Another group of proteins with great importance and also involved in biomass deconstruction is the group of enzymes known as lytic polysaccharide monooxygenases (LPMOs) classified in auxiliary activity families 9 (AA9), 10 (AA10), 11 (AA11), and 13 (AA13) in the CAZy database (Frandsen et al., 2016; Frommhagen et al., 2015; Hemsworth, Henrissat, Davies, & Walton, 2013; Silveira & Skaf, 2016; Vaaje‐Kolstad et al., 2010). From these families, AA9 corresponds to fungal proteins involved in cellulose deconstruction (some of them are also active in hemicellulose), while AA10 belongs to a bacterial group of LMPOs active on cellulose and chitin, and AA11 and AA13 are fungal proteins active on chitin and starch, respectively. AA9 proteins have been studied in *N*. *crassa* (Tian et al., 2009), *T*. *reesei* (Tanghe et al., 2015), *P*. *chrysosporium* (Westereng et al., 2011), *Chaetominium globosum* (Kim et al., 2015) and *Myceliophthora thermophile* (Frommhagen et al., 2015), and have been reported to improve the release of glucose and oligosaccharides from avicel, regenerating amorphous cellulose and lignocellulosic substrates even at a level of 150 fold increase (Frommhagen et al., 2015).

Three recent works have reported the presence of AA9 proteins in the secretomes of *S*. *commune* when cultured in avicel (one protein) (Sornlake et al., 2017), Jerusalem artichoke stalks (nine proteins) (Zhu et al., 2016), and *Leucaena leucocephala* wood chips (Singh et al., 2017) (3 proteins). This fact indicates that similar to the classic hydrolytic cellulase system, the expression of AA9 proteins in fungi is dependent on the substrate. Further analyses are necessary to evaluate the biochemical features and the position of the oxidative cleavages (C1 oxidation, C4 oxidation, or both) performed by these enzymes, since understanding the action mechanism of fungal LPMOs and gaining information about the transcriptional regulation of LPMO genes in fungi and bacteria would help to decipher how microbes fully deconstruct lignocellulosic biomass.

## 4 PROTEINS INVOLVED IN HEMICELLULOSE DECONSTRUCTION BY *SCHIZOPHYLLUM COMMUNE*

Hemicelluloses are heteropolysaccharides from the plant cell walls that constitute the second most abundant component of lignocellulosic biomass. Their complex structure is dependent on the source and mainly contains pentoses (xylose and arabinose), hexoses (glucose, galactose, and mannose) and, to a lesser extent, glucuronic and galacturonic acid. The bioconversion of hemicellulose to obtain ethanol or other value‐added products, such as chemicals and biopolymers, is a well‐researched topic. Through a pretreatment process, the hemicelluloses are degraded or broken down in the biomass, releasing fermentable sugars such as xylose, arabinose and glucose, and rendering the cellulose more accessible to cellulolytic enzymes (Lavarack, Griffin, & Rodman, 2002). Pretreatment of hemicelullose (usually chemically treated) is one of the most expensive steps of biomass processing, thus, studies to decrease the cost are of main interest from an economic point of view (Canam, Town, Iroba, Tabil, & Dumonceaux, 2013). The poor sustainability of the currently used acid/base‐catalyzed processes has highlighted the need for a more environmentally friendly and mild pretreatment of the hemicellulosic biomass, such as biological ones, that also encompasses a high efficiency (Canam et al., 2013). Another disadvantage of chemical processes is that byproducts may potentially act as microbial inhibitors during the subsequent fermentation steps (Peng, Peng, Xu, & Sun, 2012). Therefore, enzymatic pretreatment and bioconversion have arisen as a suitable alternative that could be coupled to subsequent fermentation and might enhance the industrial processing of biomass. The main drawback of enzymatic processing is that high efficiency has not been achieved to date. New enzyme cocktails that can increase the yields of fermentable products and other value‐added chemicals are currently under study (Zhu et al., 2016).

Hemicellulases are a generic family of proteins that catalyze the degradation of hemicellulosic polymers from which xylanases have been intensely researched. Xylan is the most abundant type of hemicellulose found on hardwoods and its structure is mainly (1→4)‐linked β‐d‐xylopyranosyl residues that are substituted with glucuronosyl and 4‐O‐methylglucuronosyl residues by α‐(1→2) linkages. Other substituent like acetyl, feruloyl, coumaroyl groups and α‐l‐arabinofuranose can also be of relative importance to produce the complete breakdown of hemicellulose. Generally, xylanases refer to a large group of enzymes comprising endo‐1, 4‐β‐xylanase (EC 3.2.1.8) and β‐xylosidase (EC 3.2.1.37), and several accessory enzymes with debranching activity (Peng et al., 2012). Endo‐xylanases degrade xylan at internal sites, producing xylooligosaccharides of varying length. Complementary, β‐xylosidase removes xylose residues from the end of these short oligosaccharides. Esterases are among the most studied enzymes with debranching activity on hemicellulose. Acetylxylan esterase (EC 3.1.1.72) removes the O‐acetyl of acetyl xylan, while feruloyl and coumaroyl esterases (EC 3.1.1.73) hydrolyse the phenolic compounds linked to arabinofuranoside residues. α‐l‐Arabinofuranosidase (EC 3.2.1.55) and α‐d‐glucuronidase (EC 3.2.1.139) are also responsible for the cleavage of branching structures.

The reduced capacity of *S*. *commune* to degrade the lignin components from lignocellulose has been previously reported (Floudas et al., 2015; Horisawa et al., 2015; Zhu et al., 2016) in agreement with the lack of genes encoding class II peroxidases from the AA2 family (Ohm, de Jong, Lugones, et al., 2010). Interestingly, the main enzymatic activity detected in culture supernatants from *S*. *commune* grown in lignocellulosic substrates is hemicellulolytic (Zhu et al., 2016). Nevertheless, the production of xylanase activity in this fungus is under the regulatory control of cellulosic degradation byproducts (Haltrich & Steiner, 1994). Xylan or galactomannan do not induce xylanase or mannanase activities when provided as sole carbon source. Instead, cellulose, cellobiose, lactose, and l‐sorbose induce, altogether, xylanase, cellulase, as well as mannanase activities indicating a common regulatory control in this fungus (Haltrich & Steiner, 1994).

The analysis of the genome of *S*. *commune* has shown that non‐cellulosic polysaccharide‐degrading enzymes are more abundant when compared to other model of lignocellulose decomposers (Ohm, de Jong, Lugones, et al., 2010). This fungus contains an extensive repertoire of xylan and pectin glycoside hydrolases as shown in Table 3, indicating a great potential for hemicellulose deconstruction. When compared with other basidiomycete fungi (the white‐rot *Phanerochaete chrysosporium* and *Ceriporiopsis subvermispora* and the brown‐rot *Gloeophyllum trabeum*), *S*. *commune* achieved the highest xylanase activity when growing on a lignocellulosic substrate (Zhu et al., 2016). Similarly, a crude enzymatic cocktail obtained from a solid‐state fermentation of *S*. *commune* was more effective than a commercial enzyme cocktail from *Trichoderma longibrachiatum* in terms of reducing sugar release from pretreated lignocellulosic biomass (Zhu et al., 2016). In this case, while cellulolytic activities where similar, the level of xylanases was significantly higher in the *S*. *commune* enzymatic cocktail.

**Table 3:** Hemicelulose degrading glycoside hydrolases in the genome of *S*. *commune* (modified from (Ohm, de Jong, Lugones, et al., 2010))

CAZyme family	No. Genes	Carbohydrate target	Enzyme name	No. enzymes
GH5	1	Hemicellulose	β‐mannanase	1
GH10	5	Hemicellulose	β‐1,4‐endoxylanase	5
GH11	1	Hemicellulose	β‐1,4‐endoxylanase	1
GH26	1	Hemicellulose	Glycosidase related	1
GH43	19	Pectin + hemicellulose	Exo‐b‐1,3‐galactanase	2
			α‐l‐arabinofuranosidases	12
			Glycosidase related	5
GH51	2	Pectin + hemicellulose	α‐l‐arabinofuranosidase	2
GH53	1	Pectin + hemicellulose	Endo‐β‐1,4‐galactanase	1
GH62	1	Hemicellulose	α‐l‐arabinofuranosidase	1
GH93	2	Hemicellulose	Exo‐1,5‐α‐l‐arabinanase	1
			Glycosidase related	1
GH115	2	Hemicellulose	Xylan α‐1,2‐glucuronidase	2

## 5 LIGNIN DEGRADING ENZYMES AND ALTERNATIVE BIOTECHNOLOGICAL APPLICATIONS OF *SCHIZOPHYLLUM COMMUNE*

In addition to the cellulases and xylanases studied in *S*. *commune* (Table 1), the lignin‐degrading enzymes of this fungus have also been evaluated for different biotechnological applications. According to the CAZy database, lignin‐degrading enzymes are grouped in some of the families with auxiliary activity. From these, *S*. *commune* produces only members of the AA1 (laccases; 2 genes), AA3 (cellobiose dehydrogenases: 1 gene; glucose oxidase: 4 genes; aryl alcohol oxidase: 1 gene; pyranose oxidase: 1 gene; alcohol oxidase: 1 gene) AA5 (glyoxal oxidase: 2 genes) and AA6 (benzoquinone reductase: 4 genes) families, lacking the production of lignin peroxidases (LiP), manganese peroxidases (MnP) and versatile peroxidases (VP), that belong to the AA2 family (Ohm, de Jong, Lugones, et al., 2010). Intriguingly, although *S*. *commune* does not produce MnP nor LiP as stated above, there are a variety of studies which mention that the Lip and MnP enzymes from *S*. *commune* are involved in the decolorization of *azo* and textile dyes or that the LiP and MnP from *S*. *commune* are useful enzymes for lignin removal of a variety of lignocellulosic substrates (Asgher, Wahab, Bilal, & Nasir Iqbal, 2016). It is likely that instead of MnP and LiP, the enzymes involved in the decolorization and delignification effects are members of the multi‐copper oxidases and the hydroxyl radical generation system, among others (Ohm, de Jong, Lugones, et al., 2010).

The evaluation of extracellular laccases from *S*. *commune* date to 1986 (De Vries, Kooistra, & Wessels, 1986). These enzymes are proteins with a great versatility, since they can oxidize a variety of organic and inorganic compounds, phenolic and non‐phenolic substrates, including, mono, di, polyphenols, aminophenols, and metoxyphenols (Upadhyay, Shrivastava, & Agrawal, 2016). Current laccase investigations are focused on bio‐oxidation and biotransformation processes, biosensor development, enzymatic synthesis of organic compounds, biopulping and biobleaching, textile dye transformation, removal of phenolic compounds from must and wine, waste effluent treatment, fossil fuel desulfurization, biosolubilization of coal, degradation of herbicides, food treatments, medicinal applications through the synthesis of novel compounds and delignification and biografting of lignocellulosics (Singh Arora & Kumar, 2010; Upadhyay et al., 2016). However, the search for industrial applications of *S*. *commune* ′s laccases is scarce, and is limited to dye decolorization experiments, the study of the three‐dimensional model of one of the laccases from *S*. *commune* and the activity related to delignification of lignocellulosic substrates. This lack of industrial applications of laccases from *S*. *commune* leaves open areas of studies to be exploited from various points of view.

## 6 PATENTS RELATED TO THE POTENTIAL OF *SCHIZOPHYLLUM COMMUNE* IN INDUSTRIAL APPLICATIONS

Patent databases (United States Patent Office (USPO), World Intellectual Property Organization (WIPO), European Patent Office (EPO), etc.) show an overview on technological and industrial state of the art as well as conceptual and methodological advances in the field of molecular biology and biotechnological applications (applied mycobiotechnology and myco‐remediation technology) of fungi.

In this context, there is an increase in the biotechnological significance of *S. commnune* in the last 15 years. Related to its genome, its enzymatic complexes and its biological versatility, more than 6,000 patent application documents and technological reports have been registered between 1995 and 2017 worldwide, which support *S*. *commune* as a biotechnologically functional microorganism, relevant in different technological, agricultural, environmental and pharmaceutical fields. At the EPO, Espacenet, more than 170 patent documents have been registered during 2000–2017, directly linked to technological and industrial applications of *S*. *commune*. The fields of innovation‐patentability‐biotechnology where the versatility of *S*. *commune* is currently being applied are summarized in Table 4.

**Table 4:** Granted and applied patents related with *S*. *commune*

Technical and industrial fields of the patents (applied for or granted)	Applicant(s) and year	References
Selective and oriented enzyme production and preparation		
Preparation of glucoamylase	TAX ADM Agency (Japan, 1984)	Shimazaki and Sato (1984)
Production of bilirubin‐oxidase	Takara Shuzo Co. Ltd (Japan, 1986; 1984)	Matsui, Sato, and Nakajima (1986) and Susumu, Satou, and Takako (1984)
Production of cholesterol oxidase and its use in modification of natural occurring spirostanes	Toejepast Natuur Ondersoek, (Netherland, 1988); Ono Pharmaceutical Co., Ltd. (Osaka, Japan, 1977)	Kerkenar Anthonius inventor, NO voor TNO (1988) and Sugiura, Shimizu, Sugiyama, Kuratsu, and Hirata (1977)
Production of xyloglycan endo‐transglycosylases	Novozymes A/S (Europe, 2000)	Ilum (2000)
Production of cholesterol esterase	Toyobo Co. Ltd. (Japan, 1978)	Aisui, Nakagiri, and Otawara (1976)
Production of pantolactone hydrolase	Fuji Yakuhin Kogyo Kabushiki Kaisha (Japan, 1996)	Sakamoto, Yamada, and Shimizu (1996)
Production of xylanase and laccases for treatment of wood pulp and lignin decomposition	Mercian Corp. Japan Bioindustry Association Agency of Ind. Science & Technol (Japan, 2000); Clariant Finance (bvi) Limited Sandoz (Europe, 1997)	Behrendt, Blanchette, Farrell, and Iverson (1997) and Hitoshi, Watanabe, Yoshio, and Takeo (2000)
Production of thermostable xylanases	National Research Council of Canada (Canada, 2001)	Wing (2001)
Production of thermo‐resistant trehalose phosphorylase	Kureha Chem. Ind. Co. Ltd (Japan, 2004)	Eisaki, Eiichi, Yasutake, and Toshihiko (2004)
Multifunctional cellulases	Dyadic International (USA, 2013) Ltd. (USA); Novozymes A/S (2014).	Emalfarb et al., (2013) and Kuilderd, Wu, Li, and Zhou (2014)
Enzymatic complex with chlorogenic acid esterase activity and feruloyl esterase activity	Stern Enzym GmBH & Co. KG (Denmarck, 2014)	Nieter et al. (2016)
Obtaining and preparation of secondary metabolites and derivatives with great added value		
Preparation and use of β‐glucans	Birch Stewart Kolasch & Birch (USA, 2009)	Kim, Park, and Sang‐Rin (2009)
Preparation of neoschizophyllan	Taito Co., Ltd. (Tokyo, Japan, 1978) & Kaken Chemical Co., Ltd. (Tokyo, Japan, 1978)	Kikumoto, Yamamoto, Komatsu, Kobayashi, and Kamasuka (1978)
Preparation of trehalose	Kureha Chem. Ind. Co. Ltd (Japan, 1994)	Takashi and Eisaku (1994)
Preparation of schizostatin	Sankyo Co. Ltd (Japan, 1995)	Yoshio, Kiyoshi, Tomoyuki, Tatsuo, and Takeshi (1995)
Preparation of stachyose	Infinitus (China, 2017)	Meng, Zhang, Zhou, Gao, and Duan (2017)
Obtention of ergothioneine	Mitsubishi Shoji Foodtech Co Ltd (Japan, 2015)	Tokumits (2015)
Preparation of schizophyllan	Ningbo Xinuoya Marine Biotechnology Co. Ltd (China. 2016)	Hui (2016)
Production of huperzine A	Univ. Fujian Traditional Chinese Medicine (China, 2014)	Yaxuan (2014)
Processes and prototypes		
Cosmetic creams for topical use	MAX FUAKUTAA KK (Japan)	Fukada, Kobayashi, Matsuda, Kato, Toshinori, and Kojima (1993)
Oxidative dyeing process of keratin fibers	Casalonga Axel Bureau (Europe, 2002)	Gregory (2002)
Endoglucanase treatment of lignocellulosic materials and selective degradation of resin acids and triterpenes	Novozymes, A/S (USA, 2002)	Schülein et al. (2002)
Production of II generation biofuels from vegetable biomass via cellulolytic enzymes	IFP (France, 2009)	Margeot Antoine (2009)
Process for degradation of lignin and dioxin derivatives in field conditions	Idemitsu Kosan Co. Ltd (Japan, 2001)	Yuki Junishiro (2001)
Process for degradation of exogenous endocrine disruptors	Idemitsu Kosan Co. Ltd. (Japan, 2002)	Genshi and Takahiro (2002)
Process for decomposition of prions	Kondo Ryuichiro (Japan, 2005)	Ryuichiro, Yuli, and Shiro (2005)
Process for production of alcohol or second generation solvent	IFP Énergies Nouvelles (France, 2012)	Ropars, Aymard, Guillaume, and Menir (2012)
Design of immunological cancer therapies	Therapy Co. Ltd (Japan, 2000)	Akiyuni and Takashi (2000)
Process for selective removal of hexenuronic groups from biomass	Siika‐aho, Matti (USA, 2004)	Siika‐Aho et al. (2004)
Mycelial extracts formulations for potentiating the resistance of bee colonies against fungal‐viral collapse syndrome	Paul Stamets and Co. (USA, 2015, 2017)	Stamets (2015, 2017)
Biological saccharification method using biomass	PHYGEN Inc (Korea, 2016)	Kul et al. (2017)
Nutritional additives		
Enhance immunity of lobster	Dingyuan County Profess. Coop. (China, 2016)	Guanghong (2016)
Milk cow forage	Xuzhou Jiwang Xintuo Animal Husbandry Co. Ltd. (China, 2016)	Xiume (2016)

In the field of lignocellulosic biomass, more than 1,100 patents (altogether applied and granted) related to the use of *S*. *commune* were reported in the last two decades (Gupta, 2016), including biofuel′s production and biomass derivatives. As stated before, it is recognized that economic utilization of widely distributed lignocellulosic biomass as a feedstock for the eco‐sustainable production of biocarburants, biodiesel, molecular scaffolds, biomaterials, fuels, and chemicals with high‐added value would represent a conceptual and methodological change in the strategic utilization of natural raw materials, allowing sustainable resources to be substituted for, and compete with, petroleum‐based products.

In other research areas, *S*. *commune* has also been a subject of interest. For example, a nematicidal and bacteriostatic fumigant formulation has been prepared from an *S*. *commune* strain where the main bioactive component is β‐bisabolol. This composition is environmentally friendly and shows a very wide spectrum of action (Kaiyin, 2017). Cozen Co. Ltd reported a hot water‐extracted thrombotic dissolving enzyme (9–10 kDa) from *S*. *commune* fruiting bodies, capable of being used effectively as health supplement food or a treatment agent for thrombus‐related disease (Choi Nack Shick, 2015).

**Figure 3:**
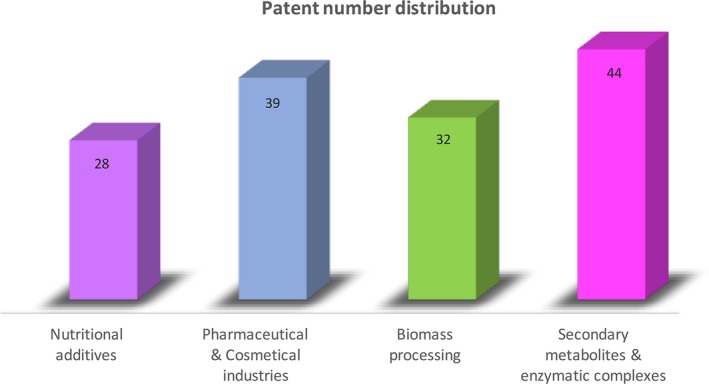
Europe Patent Office patents (1990–2017). Distribution by technological application fields

The patent database study (EPO base, 150–170 documents from 1990 to 2017) reveals that the main technological‐industrial application fields for *S*. *commune* in the last two decades were: nutritional additives for humans and animals with economic significance; agricultural biotechnology; pharmaceutical and cosmetic industry; generation of secondary metabolites with great‐added value; biotechnological application of enzyme complexes; biomass processing and bio‐refineries; and environmental issues. Some results are shown in Figure 3.

Taking this information into account, it must be highlighted that nutritional additives (nutrient feed, fermented functional beverages, forage, healthcare formulations, tonifying compositions, etc.) correspond to 20% of the overall patents reported for that period. In the case of the pharma‐cosmetic field (bioactive antibacterial‐ nematocide components, pharma compositions, extracts with selective pharmaceutical properties, anticancer and antiviral formulations, skin treatment creams, ophthalmic solutions, bio‐adhesives, anti‐oxidant and anti‐wrinkle formulations, nano‐liposomes, etc.) it corresponds to 27%. Regarding to biomass bioprocessing and related processes (bio‐pretreatment of agro‐wastes, biological saccharification, gelatin production, obtention of biofuels and bio‐derivatives at the bio‐refinery level, generation of enzymatic complexes for treatment of lignocellulosic materials and wastes, functional biofibers and bio‐oligomers, solid fermentation, pith and lignin degradation, bio‐oriented decomposition, etc.) the patents correspond to 22%, and, in the field of applied secondary metabolites, with great‐added value, and utilization of enzymatic complexes (laccases, cellulases, xylanases, esterases, oxidases, production of glucans and polysaccharides with different molecular weights, ergothioneine, schizophyllan, glucosone, xylitols, trehalose, pantolactone, retinoids, organic acids, etc.), the patents number account for 31% of the total. It is noteworthy that the observed application‐development trends will be maintained in the next 2–5 years, which supports the biotechnological versatility and applicability of this basidiomycete.

## 7 CONCLUSIONS

*Schizophyllum commune* is a fungus that has a quite complete enzymatic set that can be used for diverse areas in the biotechnological field. Its genome description as well as the recently published works and patents related to this fungus, demonstrates part of the biotechnological potential that *S*. *commune* possess. This review is the first to concentrate most of the work that has been done with *S*. *commune* in the subject of plant biomass exploitation and the enzymes involved in its degradation, with a view to its future implementation in bio‐refineries, pollutant degradation, formulation of enzymatic cocktails, bioconversion of agricultural by‐products, as an example. Additionally, *S*. *commune* is a good source for hydrolytic, non‐hydrolytic and oxidative enzymes which can help to understand the processes by which this fungus is capable of using the carbohydrates and phenolic compounds in the vast diversity of woods it can colonize, since classical genetics and genetic engineering techniques are available for *S*. *commune*.

## CONFLICT OF INTEREST

Authors declare that there are no conflicts of interest.
